# Multidimensional Benefits of Improved Sanitation: Evaluating ‘PEE POWER^®^’ in Kisoro, Uganda

**DOI:** 10.3390/ijerph17072175

**Published:** 2020-03-25

**Authors:** Jiseon You, Chad Staddon, Alan Cook, James Walker, Jess Boulton, Wayne Powell, Ioannis Ieropoulos

**Affiliations:** 1 Bristol BioEnergy Centre, Bristol Robotics Laboratory, University of the West of England, Bristol BS16 1QY, UK; jiseon.you@uwe.ac.uk (J.Y.); ioannis.ieropoulos@brl.ac.uk (I.I.); 2 International Water Security Network, Department of Geography and Environmental Management, University of the West of England, Bristol BS16 1QY, UK; alan.cook@uwe.ac.uk (A.C.); jameswalker310@gmail.com (J.W.); jessica2.boulton@live.uwe.ac.uk (J.B.); wayne2.powell@uwe.ac.uk (W.P.)

**Keywords:** Pee Power^®^, ecological sanitation, renewable energy, social acceptance, user perception, female safety, gender equality, female empowerment, sustainable development goal

## Abstract

With 2.3 billion people around the world lacking adequate sanitation services, attention has turned to alternative service provision models. This study suggests an approach for meeting the sanitation challenge, especially as expressed in Sustainable Development Goal 6.2, using a toilet technology system, such as Pee Power^®^ that generates electricity using diverted urine as a fuel. A field trial was carried out in a girls’ school in Kisoro, Uganda, where the generated electricity was used to light the existing toilet block. The trial was evaluated in terms of social acceptability and user experience using a multidimensional assessment protocol. The results of our assessment show that users felt safer when visiting the toilets at night. Lights provided from the technology also helped with the perceived cleanliness of the toilets. The technology was well accepted, with 97% of the respondents saying that they liked the idea of the Pee Power^®^ technology and 94% preferring it over other facilities on site. This shows how the technology helps meet SDG target 6.2, with its particular focus on vulnerable populations.

## 1. Introduction

Improving sanitation by eliminating open defecation (OD) and enhancing access to improved sanitation services such as toilets and handwashing facilities can prevent transmission of disease, malnutrition and stunted growth of children as well as sanitation-related morbidity and mortality [1,2,3,4]. Besides health benefits, better sanitation can lead to other benefits including improving gender equality, the welfare of the disabled and the local environment [5]. Despite a global consensus and concerted efforts at improvements, 2.3 billion people still lack even basic sanitation services such as latrines, and 673 million people practice OD with the majority of these people living in Sub-Saharan Africa, Central Asia and Southern Asia [6].

In 2015, all United Nations member states agreed to adopt the 17 Sustainable Development Goals (SDGs) which are an urgent call for action for a better and more sustainable future for all [7]. Goal 6 calls on nations to “ensure availability and sustainable management of water and sanitation for all” with eight more specific targets to be achieved by 2030, one of which (SDG 6.2) is to “achieve access to adequate and equitable sanitation and hygiene for all and end OD, paying special attention to the needs of women and girls and those in vulnerable situations”. There is considerable evidence that women and girls are at greater risk of maternal mortality, adverse pregnancy outcomes and non-partner sexual violence (NPSV) when using unimproved sanitation facilities [8,9,10,11]. Women’s stress and vulnerability in relation to sanitation provision increases even further during menstruation. This is due to a risk of feeling shame if seen or, in some cultures, through using the same facilities as non-menstruating women [12]. It is not only the lack of access to toilet facilities but also inadequate supporting services, such as poorly designed/constructed/maintained toilets or an absence of lighting, that can exacerbate these vulnerabilities [12,13]. O’Reilly has suggested the term “toilet insecurity” to describe “when safe, usable toilets are not available”, and asserts that sanitation for women and girls is closely linked to broader social structures of gender inequality [14].

Innovations in water and sanitation technologies are essential to global efforts to meet SDG 6, and the scale of the challenge is compounded by population growth. It is estimated that the global population will reach 9.7 billion people by 2050, with the largest increase coming from countries in Sub-Saharan Africa, where the sanitation challenge is greatest [15]. Not only is the global population growing, but it is also continuing to urbanize, with knock-on consequences for the proliferation of informal housing. In Uganda, areas of informal housing are characterized by extreme poverty and lack of infrastructure [16]. Such settlements in urban and rural areas are likely to be close to the bottom rung of the sanitation service ladder [17], where unimproved shared latrines and only basic sanitary protection are available. According to the latest UNICEF report on Uganda, 2.7 million people in Uganda are regularly practicing OD and only 16% of children live in homes with handwashing facilities near the toilet [18].

Pee Power^®^ is a relatively new toilet technology, developed within the context of the Bill and Melinda Gates Foundation’s “Reinventing the Toilet” program. It involves urine diversion through microbial fuel cells (MFCs) to generate electricity and resulting in a partially-treated effluent [19]. It proceeds from an ecological sanitation (EcoSan) approach, also known as ‘resource-oriented sanitation’, which is based on ecosystem approaches and the closure of material flow cycles (i.e., “circular economy”) [20]. In the EcoSan concept, human excreta and wastewater from human activities are regarded as a resource, not as waste, and the aim is to reuse the resource safely as part of an ecologically and economically sustainable wastewater management system [21,22].

When developing a technology for sanitation improvement, human elements such as social acceptance or user perception are often overlooked, increasing the likelihood of project failure. A lesson learnt from past sanitation improvement programs is that technology is only one part of the larger challenge: people’s attitudes and behaviors are critical ingredients of success or failure [23,24]. The importance of social acceptance in sanitation has been proven by the success stories emerging from rural areas that have declared themselves ‘open defecation free’ after adopting an approach called community led total sanitation (CLTS) [25,26]. It is one thing to design an alternative toilet technology and quite another for it to find acceptance amongst its intended beneficiary community.

In this study, rather than adopting a purely technologically-centered approach, we included CLTS principles such as social capacity building and community engagement, and designed the study accordingly. A new approach to tackle the sanitation issue is suggested by using a renewable energy technology to provide additional motivation for sustained toilet use. A field trial was carried out in a girls’ school in Kisoro, Uganda where some of the existing outdoor toilets were modified to integrate a Pee Power system. The electricity produced from diverted human urine was used to power lights in the toilets at night, where previously no electrical light was provided. Social acceptability and user experience as well as technical performance of the Pee Power system were evaluated using a multidimensional technology assessment framework, as proposed by others [27,28].

## 2. Methodology

In order to evaluate social acceptability and the experience of the Pee Power technology, a mixed quantitative-qualitative monitoring and evaluation (M&E) strategy was chosen. This involved the collection of both physical and social data related to the performance of the system, and the perception and use of it by its intended beneficiaries. Specifically, quantitative data from questionnaires and qualitative data through focus groups and interviews, as well as system performance data, were collected and analyzed to capture the multidimensional aspects of the field trial. Unstructured observations were also carried out throughout the year-long trial period to check for interference with, or misuse of, the system and to monitor attitudes across a meaningful span of time (i.e., beyond the “novelty phase”).

### 2.1. Study Area

**Figure 1:**
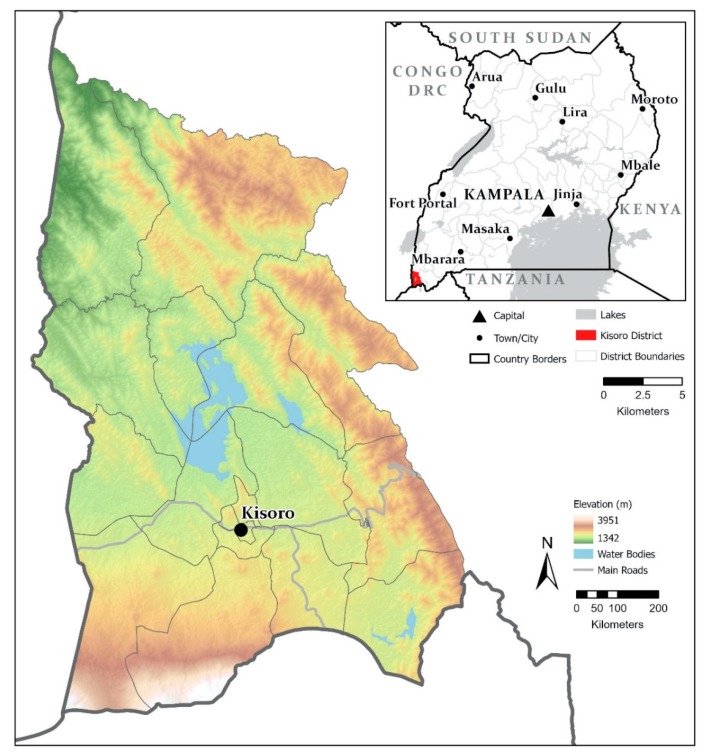
Map of the study area, located in the far southwest of Uganda (Source: Harry West, UWE, Bristol).

Kisoro is the chief town of Kisoro District, located in far southwestern Uganda, adjacent to the borders of Democratic Republic of Congo to the west, and Rwanda to the south (Figure 1). The area, at an elevation of 1890 m, is both volcanic and mountainous, with some nearby surface water (Lake Mutanda) but little available groundwater. In 2014, the town’s population was estimated at 15,859 and the district population was approximately 250,000 [29]. According to the 2014 Census, unemployment in the district of those aged 18 years and above was at 25.7% [29]. However, many jobs are unsecure and workers can go unpaid for months. The annual GDP per capita of Kisoro is estimated to be less than $200 USD [30].

This area is highly water stressed. Some urban households in the town center have a piped water supply drawn from the Chuho Spring, located around 4 km northeast of Kisoro town. However, most households outside of the town, particularly those in the more rural areas, are reliant on self-supply from various sources including untreated surface waters. It is commonly the job of women and children to fetch water. Children can be seen walking many miles in the hours before and after school carrying ‘jerry cans’ (sometimes up to 20 L in capacity and weighing 20 kg), including up steep hills. Although many roofs in the district are sufficiently large and made from galvanized iron, and may therefore be suitable for rainwater harvesting, the cost of system construction is too expensive for most and is only financially viable where local WATSAN programs are able to assist [31,32]. The topography is characterized by its volcanic nature and steep slopes, with natural water sources present in the valleys and groundwater largely inaccessible. Many people prefer to live on higher ground and avoid the valleys due to flood risk, thus putting water supply at a greater distance from the point of use.

Sanitation provision in this area is also a challenging issue. Within the Kisoro district, 4.1% households have no access to any toilet facilities at all [33]. OD is common in the villages due to the lack of sanitation facilities and the lack of sensitization to the benefits of using a latrine (where one is present). Often when a latrine is present in a community trading center, school or family compound, it lacks basic hygiene facilities such as water and soap for hand washing, or is not well-maintained or regularly cleaned. Since the ground of Kisoro district is volcanic, digging out the ground for pit toilet construction is especially difficult. In most cases, structures require a concrete base from which they are built upwards. As with rainwater harvesting infrastructure, construction of a concrete base toilet is too costly for most local households. There is no conventional mass sewerage system in place in Kisoro or the surrounding area. Soakaways and cesspits are the most common form of organized sewage management.

The year-long Pee Power field trial took place in a residential school setting and started in July 2017. The Seseme Girls’ Secondary School is a boarding school solely for girls aged between 11 and 19, located 1 km outside of Kisoro town center. At the time of the field trial, there were approximately 300 students in the school, and while many were from Kisoro and the surrounding rural areas, other students came from neighboring districts and from the capital Kampala, approximately 500 km away. There are two relatively new latrines with four cubicles apiece for the students to use. Prior to the trial, there was no electrical light installed either inside or outside of the toilets and students complained that this made them unsafe at night as they are located 50–100 m away from dormitory and study blocks along unimproved paths. School staff have access to a different latrine block, which is, unfortunately, in an even worse physical condition. The existing latrines offer no urine diversion and feed into a cesspit, which needs to be manually dug out at intervals when full. For the trial, two of four cubicles of one of the student latrine blocks were modified to accommodate urine diversion to a purpose-built shed housing the Pee Power system. LED light bulbs were installed in this toilet block (one inside each cubicle and three outside of the toilet block), powered by the Pee Power system.

### 2.2. Pee Power System Installation and Operation

**Figure 2:**
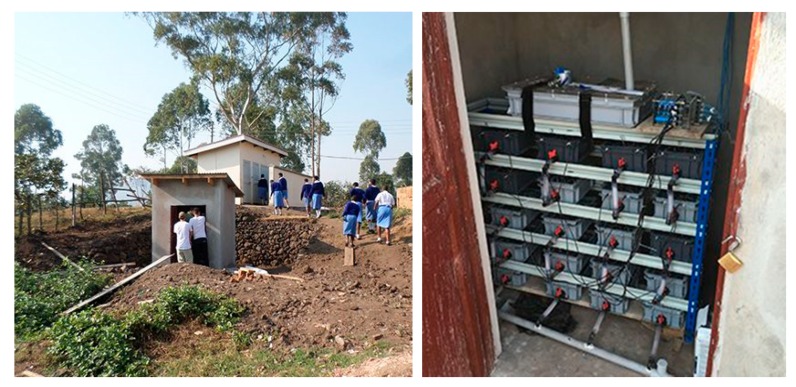
Photographs of Pee Power installation site (**left**) and system (**right**). In the left photo, building block located on the lower ground is where the Pee Power system was installed. The building on upper ground is an existing toilet block of the Seseme Girls’ Secondary School.

For installation of the Pee Power system, a concrete block structure with a corrugated iron roof was built adjacent to an existing latrine block. Diverted urine from the two existing toilet cubicles flowed to the system by gravity through a pipe. The Pee Power system comprises 20 MFC modules each containing 22 individual MFCs, an energy harvester with four rechargeable batteries and dedicated power management circuitry. In addition to operation of the LED lights, the power management system records the power generation and controls the battery charge/discharge cycle, and measures temperature and humidity in-situ, all powered by the MFCs themselves. The internal LED lights were operated with motion sensors and the external LED lights were turned on at night using a light sensor. Figure 2 shows the installation site next to the school toilets and the Pee Power system. The system was only accessible to students and staff as the school site is walled and fenced in with a watchman on the main gate. For toilet users, the most obvious aspect of service change was installation of urine diversion plates inside some toilets. Students could choose which toilet to use: either existing non-urine diverted toilets or urine diverted toilets connected to the Pee Power system.

Operation of the Pee Power system began on 19 July 2017. The system ran continuously except during occasional short disruptions, and its performance was monitored for 20 months through to March 2019.

### 2.3. Data Collection

For this study, data collection was undertaken through three common methodologies: a questionnaire survey, focus groups and logger-based monitoring of the Pee Power unit’s technical performance throughout the trial period. Key to the methodology was the need to distinguish between four potentially available outcomes:

(i) The technology works but people do not use it;
(ii) The technology does not work but people use it anyway;
(iii) The technology does not work and people do not use it; and
(iv) The technology works and people use it.

Quantitative data concerning the performance of the MFC stacks was collected from sensors to a data logger over the period of the trial. Data collected included voltage output, temperature and humidity, together with time/date stamps. The qualitative dimension of the M&E strategy was concerned primarily with user and non-user perceptions, attitudes and behaviors around the Pee Power units and involved pre- and post-installation questionnaire surveys and post-installation focus groups.

A questionnaire survey was carried out three times in total during the trial period. The first survey took place one week before the system was installed (to provide baseline data) and the second was conducted one week after installation, thus two weeks apart. These were designed to understand the baseline of user perception and attitude, as well as the initial experience of using Pee Power (the “novelty effect”). The first and second surveys had 310 and 245 respondents respectively, all of whom were students. The last survey was performed a year later (July 2018) to capture continuity and change in perception and behavior over time (there was some concern that perceptions might change once the system was no longer new or novel). A total of 234 students participated in the third survey. The questionnaires are available in the Supplementary Materials.

In addition to user perception and attitude, thoughts around sanitation and sexual violence were discussed through focus groups. The focus groups took place after the surveys, with randomly selected school students. Each focus group had 4–5 students and was facilitated by a local female social worker, who had also attended the school and had a sister currently at the school. She was also involved in local church and community groups, and all the girls in the groups knew her well. It was important that all of the participants were comfortable speaking with her, since the group covered sensitive issues such as toilet use and the aim was to make the discussion as open as possible. The focus groups lasted 1–2 h and were held in the evening after classes, in school classrooms. Conversations were voice recorded and notes were taken by the facilitator. Some of the questions asked in the group discussions are listed in Table 1.

Lastly, the performance of the Pee Power system in terms of electrical energy production and light operation was monitored using a custom-built energy harvester with data logging module. Temperature and humidity were also monitored during the trial, as these environmental parameters could influence the system performance.

### 2.4. Research Ethics

This study was approved under UWE Bristol’s Research Ethics procedures (approval no. 12/YH/0493) and local approval was obtained from the Seseme Girls’ School Academic Administration, in conjunction with the Diocese of Muhabura, which owns and manages the school. Informed consent was received from all respondents, whose identities are anonymized in line with common research practice.

## 3. Results and Discussion

### 3.1. System Performance

**Figure 3:**
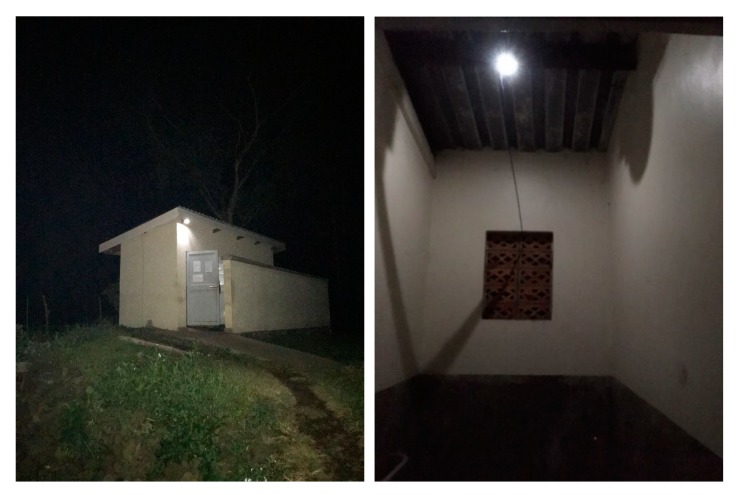
Photos of lights powered by the Pee Power system outside of a latrine block (**left**) and inside a toilet cubicle (**right**).

Overall, operation of the Pee Power system was successful in achieving the primary goal of providing lighting outside the latrine block and inside each serviced cubicle (Figure 3). Before Pee Power, these were in total darkness at night.

**Figure 4:**
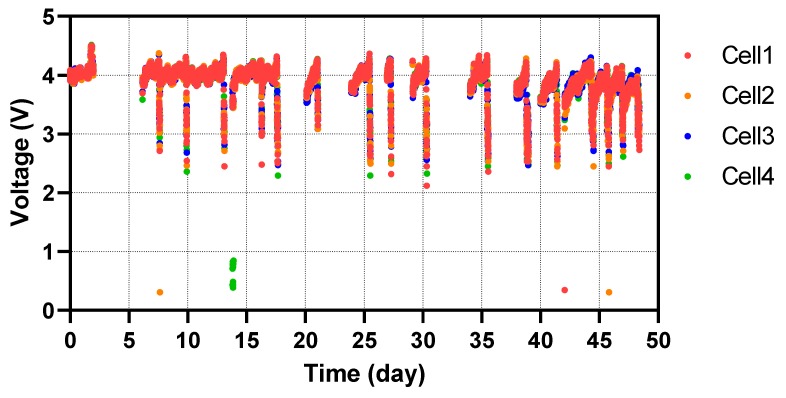
Pee Power system performance monitored between 19 July and 8 September 2017.

Figure 4 shows the charging/discharging cycles over a 50-day period of the four rechargeable batteries (used in sequence) for powering the LED lights during night time, indicating that MFC modules were producing sufficient power to maintain this operation.

A few unexpected technical challenges were encountered during the field trial. The system operation was interrupted several times during the 20-month operational period when the connecting pipe between the toilets and system was damaged by free-grazing animals (trampling), or was blocked by litter that had made it past the urine diversion plates. However, the system quickly recovered once these issues were resolved and continued powering the lights until the end of the trial. Moreover, the distinctive robustness of the technology was proven when the system restarted after a two-month school holiday, during which the school was closed and the toilets were not used at all. This implies that the MFCs can autonomously regenerate after having been completely starved and dehydrated, once urine flow is restored.

### 3.2. Safety Challenge

**Figure 5:**
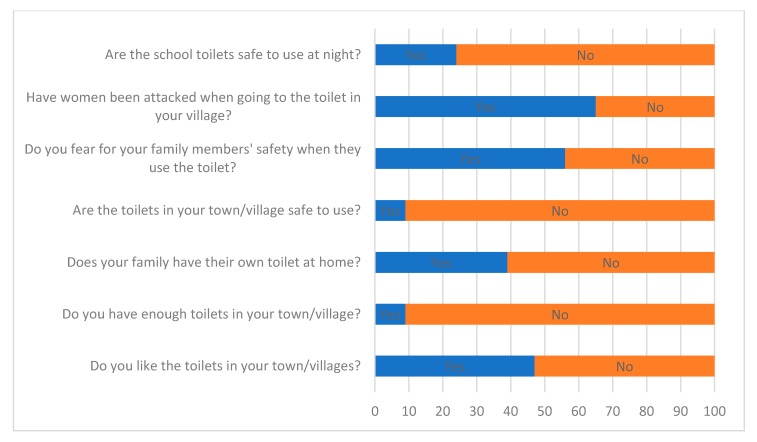
Pre-installation questions and answers.

Pre-installation surveys revealed people’s thoughts on the sanitation situation in their villages and in the school. As previously mentioned, there were already four cubicles in each of two latrine blocks for students on the school premises. Most students use these toilets during the day, but usage of the facilities drops at night and a number of girls often chose to practice OD at night rather than make the unlit journey to the conventional toilet blocks. As shown in Figure 5, 76% of respondents thought that the non-Pee Power toilets were not safe to use at night. The latrine block closest to the student dormitories is four meters from the school fence, which had recently been repaired due to an attempted break in by intruders. The wire fence was also damaged in places where intruders have lent through it to speak to the girls and offer them gifts (there are signs around the school warning against ‘sugar daddies’). There have also been recorded cases of assault and rape within the school grounds in the past three years. The danger and fear of male attack leads some girls to use the latrines at night only in groups, to better ensure their safety. Several girls would only enter the toilet cubicles if a friend or peer would wait outside, and they would usually swap once finished.

Not only did the conventional toilets in the school not feel safe but over 90% of the girls said the toilets in their villages were not safe. Many reported that women had been injured when using toilets in their villages, though this was not always attributable to male violence. The focus groups highlighted several reasons for this, including slippery (dirty) toilet floors, trip hazards, animal or insect bites, but, most importantly, attacks by men. In fact, 65% of the respondents were aware that women had been attacked by men in their villages whilst using the toilet. Understandably, it was observed that most of the female students were very cautious when a male stranger (including members of the Pee Power engineering team) was around.

**Figure 6:**
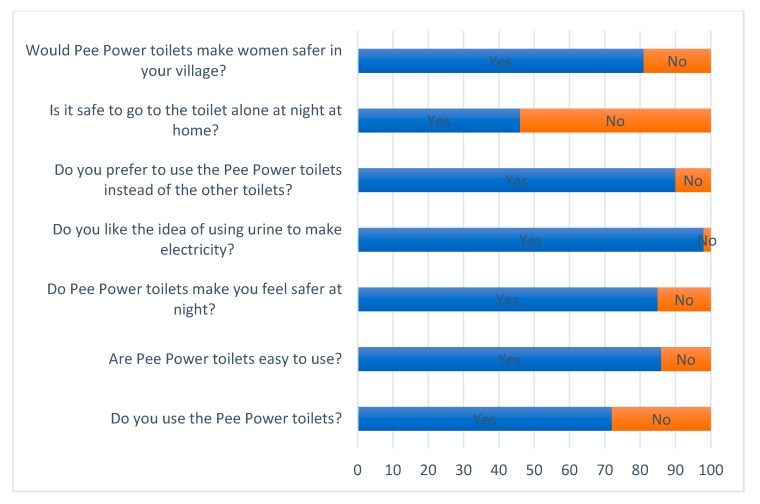
First post-installation (1 week of operation) questions and answers.

**Figure 7:**
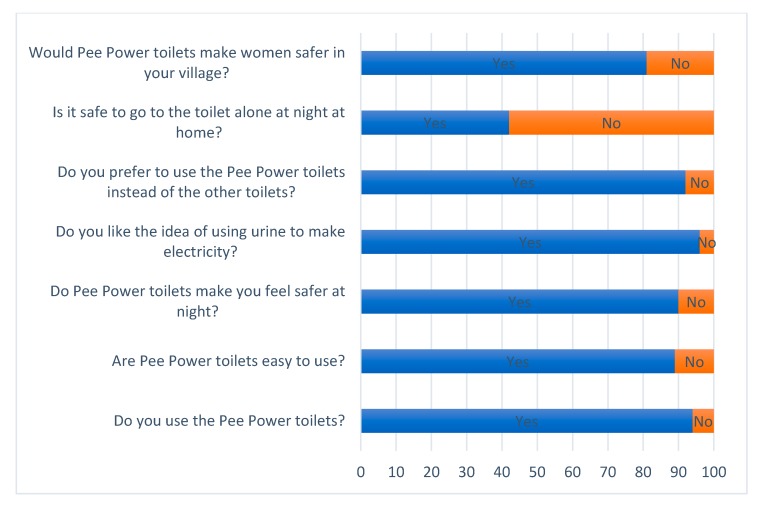
Second post-installation (1 year of operation) questions and answers.

The 1st and 2nd post-installation surveys (Figure 6 and Figure 7), carried out one year apart, show how the Pee Power technology helps users to feel safer. Immediately after inauguration of the Pee Power toilets, 85% of school students said that the lights powered by Pee Power made them feel safer when using the toilets at night. The majority of girls (82%) thought Pee Power toilets would make women safer in their villages, due to the lighting. The 2nd post-installation survey was carried out one year after the commissioning of the Pee Power toilets and showed very similar results in terms of safety (Figure 7). Of course, *feeling* safe and *being* safe are not the same. However, several studies have reported that the lack of light in latrine cubicles is one of the reasons why women fear using toilets at night [12,16]. And as Cooper et al. pointed out [34], this particular fear is a female stress factor around the sanitation issue generally. Additionally, according to the Headteacher of the school, there were no reported incidents of intrusion or assault on the pupils during the time that Pee Power was running at the school (personal communication, 21 February 2019). Therefore, providing safety by a relatively simple solution (i.e., MFC-powered lighting) is worth pursuing, especially with a renewable energy technology such as Pee Power. Focus group participants noted: “I like the Pee Power toilets because I could see a man hiding at night”, and: “Pee Power toilets are safer than the other school toilets because of the lights. With them around the toilet block it is bright enough to see if any strangers are present”.

One notable finding is that immediately after installation, 55% of respondents still felt that it was not safe to go to the toilet alone at night (Figure 6). One of the reasons was that the path from the dormitories to the toilet block (roughly 50 m) was still unlit, which was clearly far too long a walk in the dark for many of the girls. Unfortunately, this question was not asked in the third survey.

### 3.3. Sanitation Challenge

According to the sanitation service ladder used for SDG monitoring [35], the bottom rung of the sanitation ladder is where neither improved nor unimproved forms of sanitation facilities are available, making OD the only possible option. One step up is where unimproved sanitation facilities such as pit latrines without a slab or platform, or bucket latrines are available. The next rung on the ladder is where improved facilities are provided but are shared with other households. At these limited service levels, people may still choose not to use available facilities, and instead resort to OD. There are a number of reasons for this failure. Toilets that are poorly managed due to a lack of resources or education on how to use them properly, can quickly become physically unusable or undesirable [16,36]. Feelings of shame, fear of harassment or potential exposure to NPSV can be major issues for women [11,12,13]. If the toilets are privately owned and also someone’s source of income, affordability might be another reason to seek alternatives including OD [37]. Therefore, these aspects should be approached from multiple angles and dealt with through a holistic approach such as CLTS.

At the beginning of this project, only 38% of respondents said they had individual household latrines and 91% of respondents were aware of the lack of toilet facilities in the villages (Figure 5). Only 46% of students said they liked the existing toilets in their town or villages. Focus group participants said that shared toilets were usually badly maintained and thus dirty, and they did not want to be seen using these toilets because it is ‘embarrassing’ as well as potentially unsafe.

In both post-installation surveys (Figure 6 and Figure 7), most students (91% in the first post-installation survey and 94% in the second) said that they preferred to use the two cubicles that had the Pee Power system connected over the other toilets in the same block which drained directly into a septic tank. As a result, the proportion of students who were Pee Power users increased from 71% to 94% over the course of the field trial, indicating the increase of acceptability of the technology by users. Likability of the Pee Power working principle was exceptional, with almost all of the respondents (98% in the first post-installation survey and 97% in the second) saying that they liked the idea of the Pee Power technology. In fact, this high level of interest became ‘teachable’ in the context of school science and technology lessons, which were subsequently developed and delivered by the study team.

Besides improving safety, study participants have informed us that the toilets were cleaner because they could see inside the cubicles better at night: “Pee Power toilets are easier to use because we can see the toilet holes and aim properly”, and: “I used to take a torch at night and it was hard to hold a torch whilst relieving myself. It was also too expensive to buy batteries.” Moreover, students saw the potential financial benefit of the system, and even wondered if the electricity produced by the toilets could bring down their school fees. Since Pee Power did not replace existing energy use, but created a new resource, it was never going to reduce school fees or facilities running costs. Nonetheless a broader roll-out of such technologies could create an economic benefit, possibly through inspiring a new value chain. Tumwebaze et al. reported that uptake of EcoSan toilets in Uganda was associated with awareness of their economic value, which made them more attractive to users, consequently contributed to increasing coverage [38]. Therefore, it would be possible to use technology, especially renewable technology, as an economically sustainable tool for successful sanitation interventions.

### 3.4. Advancements in Girl’s Education

Besides the aforementioned benefits of the approach which combines the sanitation challenge with sustainable energy technology, there was another potential benefit that needs to be emphasized. Since the trial was carried out on the school’s grounds, we observed a great level of interest in the Pee Power technology from the students. During several visits to the site, many students approached the team to ask how the system works and what more can be done with the electricity produced by the technology. At the time they were also planning the founding of a science club to find out more about this and other technologies. According to the Headteacher (personal communication, February 2018) the number of new admissions to Seseme School increased significantly in the period that Pee Power was running. The toilets were featured in local newspapers and, although anecdotal, the school authorities felt that the two phenomena were linked.

Studies have shown that there is a distinct and positive correlation between improved sanitation and enhancement of girls’ educational outcomes including school attendance and academic performance [39,40,41,42]. When further development of the technology is made, providing more electricity to the school will be feasible, which then can be used for helping the students study at night or during the frequent interruptions of mains electricity. This may in turn lead to attracting larger numbers of students, an improvement of their learning outcomes, and eventually the economic and social empowerment of more girls in the area. Verbal reports from the school’s administration and academic staff have confirmed that those girls going to the toilet at night would prefer to stay by the light just outside the toilet block (mounted on the toilet block structure, overlooking the hill) simply because it would stay on all through the night and therefore was creating an environment in which the girls could socialize and study. The Pee Power team plans to extend lighting back towards the classroom and dormitory blocks.

The importance of the role of women in the sanitation sector cannot be underestimated. A study discussing the challenges and needs of women in relation to the water and sanitation sector has highlighted the importance of having female professionals and extension service workers on the frontline since women respond better to women than men on these issues [43]. Women should be equal users of the services in terms of accessibility and frequency of use. In some ways, the role of women is more important than that of men, since women have more physical contact with other household members, especially babies and young children, and therefore a greater impact on their health. Additionally, they are usually the ones who pass their practices on to children. Psychosocial stress linked to sanitation is much higher amongst women in comparison to men [36,44], in particular during menstrual or pregnancy periods. They often wait until very late at night or wake up early (before men) to relieve themselves. Moreover, sexual harassment is prevalent and there is a potential risk of sexual assault and rape [45,46,47], all of which put women in a vulnerable position. However, even though there is a large body of literature and fora on this subject, women have not yet been as active as one would have anticipated. There are a number of social, cultural and religious barriers to overcome in this context, and because of this complexity, multidimensional approaches should be pursued. Just as 193 United Nations states agreed on the necessity of gender equality and empowerment of all women and girls for our sustainable future (SDG 5) [7], we do hope our trial becomes a stepping stone on their journey, by improving safety and offering a learning platform of technology.

## 4. Conclusions

In this study, an approach to tackle the sanitation challenge was tested by introducing a toilet technology, Pee Power^®^, to an educational setting. A one-year field trial was carried out in a girls’ secondary school in Kisoro, Uganda. A multidimensional assessment of the trial revealed high levels of social acceptance and a very positive user experience of the technology, with over 90% of school students saying they prefer to use the Pee Power toilets over other toilets. The results of our assessment show that female toilet users feel safer at night owing to the free lighting provided through the Pee Power technology. This lighting made the facility easier to use and made users feel less vulnerable to human and animal threats. Technically, the Pee Power units performed well, though the trial revealed some design and operation issues that need to be incorporated into guidance. Beneficiaries were enthusiastic and curious about the technology and wanted to see it rolled out more widely in their communities.

The findings of this study can inform the developers on the next cycle in the technology design process—such that future designs, where possible, can conform to social needs and expectations and deliver multiple co-benefits linked not just to sanitation but also to gender inclusivity and to equality and sustainable infrastructure.

## Figures and Tables

**Table 1:** Example questions used for focus groups.

Question Type	Questions
**Introductory**	Can you please tell us about your experiences of using the Pee Power toilets? Can you please tell us whether you or someone that you know has ever experienced any injury, harassment or sexual violence while going to the toilet?
**Transition**	Can you tell us about what you like and do not like about Pee Power toilets? Do you feel safe when using the toilet?
**Focus**	What are the greatest sanitation needs of the community? What can be done to lower harassment or violence suffered while using the toilet? How can Pee Power toilets help achieve this?
**Summarising**	Thinking about today’s discussion, how do you think Pee Power toilets can be improved to make using the toilet safer?
**Concluding**	Is there anything else that anyone would like to add that hasn’t been discussed?

## Supplementary Materials

The following are available online at https://www.mdpi.com/1660-4601/17/7/2175/s1, Document S1: Questionnaire round 1, Document S2: Questionnaire round 2, Document S3: Questionnaire round 3.
