# Supplementary material for: Multidimensional Benefits of Improved Sanitation: Evaluating ‘PEE POWER®’ in Kisoro, Uganda
Source: Int J Environ Res Public Health. 2020 Mar 25;17(7):2175. doi: 10.3390/ijerph17072175 (PMC7177953; doi:10.3390/ijerph17072175)
Supplement: Supplementary file 1 [file ijerph-17-02175-s001.zip › Questionnaire round 1.pdf]

BRISTOL

1. Are you male or female? I am male / I am female
2. Are you under 16 years old or over 16 years old? I am under 16 / I am over 16
3. Do you like the toilets in your village? Yes / No
4. Do you have enough toilets in your village? Yes / No
5. Are the toilets in your village safe? Yes / No
6. Do you fear for your family member’s safety when they use the toilet? Yes / No
7. Are the school toilets safe to use at night? Yes / No
8. Does your family have their own toilet? Yes / No
9. Has a women been attacked when going to the toilet in your village? Yes / No
