# Supplementary material for: Multidimensional Benefits of Improved Sanitation: Evaluating ‘PEE POWER®’ in Kisoro, Uganda
Source: Int J Environ Res Public Health. 2020 Mar 25;17(7):2175. doi: 10.3390/ijerph17072175 (PMC7177953; doi:10.3390/ijerph17072175)
Supplement: Supplementary file 1 [file ijerph-17-02175-s001.zip › Questionnaire round 2.pdf]

1. Are you Male or Female?	I am male / I am female
2. Are you under 16 years old or over 16 years old?	I am under 16 / I am over 16
3. Do you use the Pee-power toilets?	Yes / No
4. Are Pee-power toilets are easy to use?	Yes / No
5. Do Pee-power toilets make you feel safer at night?	Yes / No
6. Do you like the idea of using urine to make electricity?	Yes / No
7. Do you prefer to use the Pee-power toilets instead of the other toilets?	Yes / No
8. Is it safe to go to the toilet alone at night?	Yes / No
9. Would Pee-power toilets make women safer in your village?	Yes / No
