# Supplementary material for: Multidimensional Benefits of Improved Sanitation: Evaluating ‘PEE POWER®’ in Kisoro, Uganda
Source: Int J Environ Res Public Health. 2020 Mar 25;17(7):2175. doi: 10.3390/ijerph17072175 (PMC7177953; doi:10.3390/ijerph17072175)
Supplement: Supplementary file 1 [file ijerph-17-02175-s001.zip › Questionnaire round 3.pdf]

Survey of Experience with Pee Power Toilet at Sesame School

February/March 2018

1. Basic Information

1. Are you male or female? I am male / I am female

2. Are you under 16 years old or over 16 years old? I am under 16 / I am over 16

2. Sanitation Experiences

3. Do you like the toilets in your town/village? Yes / No

4. Do you have enough toilets in your town/village? Yes / No

5. Does your family have their own toilet at home? Yes / No

6. Are the toilets in your town/village safe to use? Yes / No

7. Do you fear for your family members' safety when they use the toilet? Yes / No

8. Have women been attacked when going to the toilet in your village? Yes / No

9. Are the school toilets safe to use at night? Yes / No

3. Experiences with "Pee Power"

10. Do you use the Pee Power toilets? Yes / No

11. Are Pee Power toilets easy to use? Yes / No

12. Do Pee Power toilets make you feel safer at night? Yes / No

13. Do you like the idea of using urine to make electricity? Yes / No

14. Do you prefer to use the Pee Power toilets instead of the other toilets? Yes / No

15. Would Pee Power toilets make women safer in your town/village? Yes / No

4. Looking forward

16. Would you like to see more Pee Power toilets in your community? Yes / No

17. What else would you like to tell us about your experience with Pee Power toilets?
